# Lack of Association Between Dopamine Beta-Hydroxylase (DBH) 19-bp Insertion/Deletion Polymorphism and Risk of Schizophrenia 

**Published:** 2016-10

**Authors:** Mansour Shakiba, Mohammad Hashemi, Sara Shahrabadi, Maryam Rezaei, Mohsen Taheri

**Affiliations:** 1 Health Promotion Research Center, Zahedan University of Medical Sciences, Zahedan, Iran.; 2 Department of Psychiatry, Zahedan University of Medical Sciences, Zahedan, Iran.; 3 Department of Clinical Biochemistry, School of Medicine, Zahedan University of Medical Sciences, Zahedan, Iran.; 4 Cellular and Molecular Research Center, Zahedan University of Medical Sciences, Zahedan, Iran.; 5 Genetics of Non-Communicable Diseases Research Center, Zahedan University of Medical Sciences, Zahedan, Iran.

**Keywords:** *Deletion*, *Dopamine Beta-Hydroxylase*, *Insertion*, *Polymorphism*, *Schizophrenia*

## Abstract

**Objective:** Interaction between genetic and environmental factors is considered as major factors in Schizophrenia (SCZ). It has been shown that dopaminergic and noradrenergic neurotransmission dysfunction play an essential role in the SCZ pathogenesis.

This study aimed to find the impact of functional 19-bp insertion/deletion (ins/del) polymorphism in dopamine beta-hydroxylase (DBH) gene on SCZ risk in a sample of Iranian population.

**Method:** This case-control study was conducted on 109 SCZ patients and 116 matched healthy subjects. Genomic DNA samples were extracted from peripheral blood cells using salting out method. Genotyping of 19-bp ins/del DBH polymorphism was done using Polymerase Chain Reaction (PCR) method.

**Results:** Neither the overall chi-square comparison of cases and controls (𝜒2 = 0.56, p = 0.756), nor the logistic regression analysis (which was computed in codominant, dominant and recessive model of inheritance) showed any association between DBH 19-bp I/D and SCZ in a sample of southeast Iranian population.

**Conclusion**: Overall, our results did not support an association between DBH 19-bp I/D polymorphism and risk/protection of SCZ.

Schizophrenia (SCZ) is a serious mental disorder characterized by a breakdown of thinking and emotions, with a loss of contact with reality (1). It affects approximately 1% of the general population worldwide (2). It has been proposed that both genetic and environmental factors play an essential role in disease incidence and occurrence while genetic factors are responsible for up to 80% of SCZ cases (3). 

Dopamine beta-hydroxylase (DBH) is an intracellular enzyme that catalyzes the conversion of dopamine to norepinephrine (4). DBH knockout mice lack noradrenaline and show attenuated dopamine levels in various brain areas due to interactions between noradrenergic and dopamine systems (5). Dysregulation of dopaminergic and noradrenergic system has been considered to contribute to the pathogenesis of SCZ (6-11). The DBH gene is mapped on the long (q) arm of chromosome 9 at position 34 (9q34) and contains 12 exons with approximately 23 kb in length (12). The protein level of DBH is under strong genetic control and has a significant association between its activity in plasma and cerebrospinal fluid (CSF) (13-15). Low DBH activity has been proposed as a biological marker for schizophrenia and other psychiatric disorders (16). 

A 19-bpins/del polymorphism, located 4.5 kb upstream of the transcriptional start site of DBH gene, has been shown to have a strong association with DBH enzymatic activity (16, 17). A significant correlation between the DBH enzymatic activity and 19-bp ins/del variant of DBH gene has been reported (18). The minor allele (del allele) is associated with decreased promoter activity causing lower DBH levels and enzyme activity in plasma and cerebrospinal fluid (CSF) (16, 19). Low levels of DBH in the plasma or cerebrospinal fluid (CSF) are associated with greater susceptibility to positive psychotic symptoms in several psychiatric disorders (17(.

Some studies have evaluated the impact of 19-bp ins/del polymorphism of DBH gene in SCZ (20-23). To the best of our knowledge, no data are available on the impact of DBH gene polymorphism and the risk of SCZ in Iranian population. Therefore, this study aimed to find the possible association between 19-bp ins/del polymorphism of the DBH gene and risk of SCZ in a sample of southeast Iranian population.

## Materials and Method

This case-control study was done from January 2014 to June 2015 on 109 unrelated schizophrenic patients who referred to Baharan Psychiatric Hospital (Psychiatric hospital of Zahedan University of Medical Sciences). One hundred sixteen age and sex matched normal subjects free from any signs of neuropsychiatric disorders and unrelated to each other and the patients were selected as controls. SCZ diagnosis was based on DSM-IV-TR (Diagnostic and Statistical Manual of Mental Disorders, Fourth Edition, Text Revision) Criteria. 

**Figure1:**
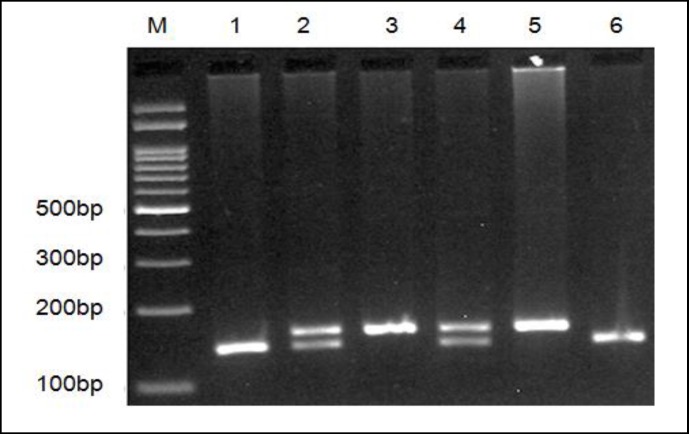
Electrophoresis Pattern of PCR Product of 19-bp ins/del Polymorphism of DBH Resolved by 2.5% Agarose Gel Electrophoresis. M: DNA Marker; Lanes 1, 6: del/del; Lanes 2, 4: ins/del; Lanes 3, 5: ins/ins

The local Ethics Committee of the Zahedan University of Medical Sciences approved the project, and written informed consent was obtained from all participants. Two milliliter of venous blood was drawn from each participant into EDTA, containing tube and genomic DNA, and extracted using salting out protocol; the samples were stored at -20 ˚C until use (24). Genotyping of DBH 19-bp ins/del was performed by polymerase chain reaction (PCR) method (17). The forward 5`-GCAAAAGTCAGGCACATGCACC-3` and reverse 5`-CAATAATTTGGCCTCAATCTTGG-3` primers were used to generate fragments of 141-bp (del allele) or 160-bp (ins allele). In each 0.20 ml PCR reaction tube, 1 µl of genomic DNA (~100 ng/ml), 1 µl of each primers and 10 µl of 2X Prime Taq Premix (Genet Bio, Korea) and 7-µl ddH2O were added. The PCR cycling conditions were set as follows: 95˚C for 5 min, 30 cycles of 95˚C for 30 s, 63˚C for 30 s, and 72 ˚C for 30 s and a final extension step of 72 ˚C for 10 min. The PCR products were separated by electrophoresis on a 2.5% agarose gel containing 0.5 μg/mL of ethidium bromide followed by transillumination with ultraviolet light and finally were digitally imaged for genotyping (Figure 1). To confirm genotyping quality, we regenotyped approximately 20% of the random samples and the concordance rate was 100%.

***Statistical Analysis***

Statistical analysis was done by statistical package SPSS 18 software. Data were analyzed using independent sample t-test or χ2 test according to the data. Odds ratio (OR) and 95% confidence intervals (95% CI) were calculated from logistic regression analyses to determine the possible association between the variants and ALL. A p-value of less than 0.05 was considered as statistically significant.

## Results

The study group consisted of 109 SCZ patients (79 males, 30 females; age: 35.7±9.8 yrs.) and 116 healthy participants (86 males, 30 females; age: 33.3±12.3 yrs.). No significant difference was detected between the groups regarding age (p = 0.134) and sex (p = 0.88(.

**Table1:** Genotype and Allele Frequencies of the DBH 19-bp Insertion/Deletion (ins/del) Polymorphism in Schizophrenics and Healthy Controls

**DBH 19-bp ins/del**	**Cases** **n (%)**	**Controls** **n (%)**	**OR (95%CI)**	**p**
**Codominant**				
**del/del**	30 (27.5)	30 (25.8)	1.00	-
**ins/del**	51 (46.8)	59 (50.9)	0.86 (0.46-1.62)	0.748
**ins/ins**	28 (25.7)	27 (23.3)	1.04 (0.50-2.16)	0.949
**Dominant**				
**del/del**	30 (27.5)	30 (25.8)	1.00	-
**ins/del+ins/ins**	79 (72.5)	86 (74.2)	0.92 (0.51-1.66)	0.917
**Recessive**				
**del/del+ins/del**	81 (74.3)	89 (76.7)	1.00	-
**ins/ins**	28 (25.7)	27 (23.3)	1.14 (0.62-2.09)	0.757
**Allele**				
**del**	111 (51.0)	119 (51.3)	1.00	-
**ins**	107 (49.0)	113 (48.7)	1.02 (0.70-1.47)	0.968

ins: insertion; del: deletion

Table 1 demonstrates the genotype and allele frequencies of DBH 19-bp ins/del polymorphism in SCZ patients and healthy participants. The findings revealed that neither the overall chi-square comparison of the cases and controls (𝜒2 = 0.56, p = 0.756), nor the logistic regression analysis (which was computed in each model of inheritance) proved any association between DBH 19-bp I/D and SCZ incidence [codominant (OR = 0.88, 95% CI = 0.47-1.66, P = 0.746, ins/del vs. del/del; OR = 1.13, 95% CI = 0.53-2.38, p = 0.849, ins/ins vs. del/del), dominant (OR = 0.95, 95% CI = 0.53-1.76, P = 0.987, ins/del+ins/ins vs. del/del) and recessive (OR = 1.22, 95% CI = 0.66-2.29, P = 0.633, ins/ins vs. del/del+ins/del)]. In addition, no significant association was observed between the insertion allele and SCZ (OR= 1.06, 95% CI = 0.73-1.54, P = 0.848). 

The genotype frequency of the DBH19-bp ins/del variant was tested for Hardy-Weinberg equilibrium (HWE) in the cases and controls separately. The genotypes in cases (𝜒2 = 0.445, p = 0.504) and controls (𝜒2 = 0.096, p = 0.756) were in HWE.

## Discussion

Dysfunction of the dopaminergic and noradrenergic systems has already been well documented to be associated with SCZ (25). The genetic association between DBH and SCZ has been investigated in numerous previous studies (26). In this study, our finding indicated that DBH 19-bp ins/del gene polymorphism was not associated with the risk of SCZ in the selected population in the southeast population of Iran. 

In agreement with our findings, Hui et al. (20) found no significant association between DBH 19-bp ins/del polymorphism and SCZ in Chinese population. Zhuo et al. (21) investigated DBH 19-bp ins/del gene polymorphism in SCZ patients with and without tardive dyskinesia (TD) and healthy controls in Chinese population. They found no significant differences in genotype and allele frequencies between the patients and controls or between the patients with and without TD. Yamamoto et al. (22) findings did not support an association between DBH 19-bp ins/del gene polymorphism and risk of SCZ. A meta-analysis performed by Dai et al. (27) revealed that DBH 19-bp ins/del gene polymorphism was not a risk factor for SCZ susceptibility. Hui et al. (28) reported that the allelic and genotypic frequencies of the DBH 19-bp ins/del polymorphism were not significantly different between schizophrenic patients and healthy controls. The cognitive test scores were significantly lower in patients than in healthy controls. They found that the attention score significantly differed according to the genotypic group in patients, but not in the healthy controls.

It has been shown that the del allelic and genotypic frequencies of DBH were significantly lower in controls than in patients with the first-episode of schizophrenia (FES), but controls were not significantly different from chronic schizophrenics (29(.

DBH plays an important role in the noradrenergic system. This enzyme is located within vesicles of central noradrenergic and adrenergic neurons and neurosecretory cells where it catalyzes the conversion of DA to NE (30, 31). The DBH 19-bp ins/del variant has been found to have a strong association with promoter activation and plasma DBH activity (16). A significant correlation was found between the DBH enzymatic activity and 19-bp ins/del variant of DBH gene (18). It has been shown that DBH variant was associated with susceptibility to psychotic symptoms in SCZ (21, 29 and 32). A decline in the DBH activity elevates the DA/NE ratio in the brain, which may contribute to the disease.

Recently, Long et al. (33) have found that DBH rs1611114 polymorphism is associated with SCZ susceptibility and related clinical symptoms in the Chinese, but not in Han Chinese population. 

The discrepancy in findings between studies may be due to genetic and environmental variances among the different populations being investigated.

## Limitations

There are some limitations in the current study. One of the limitations of this study is its relatively small sample size, so we could not perform subgroup analyses. Another limitation of our study is that we investigated only one variant of DBH gene. Thus replication with larger sample size and other variants of DBH is highly recommended.

## Conclusion

In conclusion, the findings of this study showed that a 19-bp ins/del functional polymorphism of DBH gene did not affect the risk of schizophrenia in a sample of Southeast Iranian population. Conducting similar studies with larger sample sizes and different ethnic populations are needed to confirm the findings.
